# Supplementary material for: SpikeLab: Agentic tools for spike data analysis
Source: bioRxiv. 2026 Apr 29:2026.04.25.720833. Preprint. [Version 1] doi: 10.64898/2026.04.25.720833 (PMC13142478; doi:10.64898/2026.04.25.720833)
Supplement: Supplement 2 [file NIHPP2026.04.25.720833v1-supplement-2.pdf]

bioRxiv preprint doi: https://doi.org/10.64898/2026.04.25.720833; this version posted April 29, 2026. The copyright holder for this preprint (which was not certified by peer review) is the author/funder, who has granted bioRxiv a license to display the preprint in perpetuity. It is made available under aCC-BY 4.0 International license.

Supplementary materials

Spikesorting module

SpikeLab includes a spike sorting module (`spikelab.spike_sorting`) that provides a text-to-analysis interface for the spike sorting stage of the electrophysiology pipeline. Built on top of SpikeInterface[1], the module supports three sorting backends, Kilosort2[2], Kilosort4[3], and RT-Sort[4], and implements a multi-stage curation workflow that can filter units based on firing rate, ISI violation ratio, signal-to-noise ratio, spike count, and waveform spatial consistency. A dedicated spike-sorter skill wraps this module for agentic access, extending the text-to-analysis framework from the analysis stage back to the data preprocessing stage.

Sorting backends

The module implements a backend abstraction layer (`SorterBackend`) that defines three operations: load and preprocess a recording, run the sorter, and extract per-unit waveforms. Each backend maps these operations to a specific sorting algorithm while exposing a unified interface to the pipeline.

Kilosort2 is a template-matching algorithm that requires MATLAB. SpikeLab supports two execution modes: a local installation with a user-provided Kilosort2 source path, and a Docker container that bundles the compiled MATLAB Runtime (no MATLAB license required). The Docker image is selected automatically based on the host GPU's NVIDIA driver version.

Kilosort4 is a pure-Python implementation using PyTorch. It runs locally with a CUDA-enabled PyTorch installation or in a Docker container. Kilosort4 uses updated template-matching and clustering algorithms compared to Kilosort2 and requires no MATLAB dependency.

RT-Sort is a deep-learning-based sorter that detects spikes using a trained neural network model and clusters them based on propagation sequence similarity across the electrode array. RT-Sort requires PyTorch with CUDA and is not available through Docker. The trained RT-Sort object is serialized to disk after sorting, enabling reuse in stimulation-aware sorting (see below).

All three backends produce SpikeInterface-compatible sorting objects, which are then processed through the same downstream pipeline: waveform extraction, SpikeData construction, and curation. The backend architecture is designed for extensibility. Adding a new sorting algorithm requires only implementing the sort step itself; everything else (recording loading, configuration management, format detection, folder setup, logging, waveform extraction, SpikeData conversion, curation, figure generation, compilation, and pickle serialization) is handled by the shared pipeline and applies automatically to any registered backend.

Recording handling

The module accepts recordings in Maxwell HDF5 (`.raw.h5`) and NWB (`.nwb`) formats natively. In addition, any pre-loaded SpikeInterface `BaseRecording` object can be passed directly as

bioRxiv preprint doi: https://doi.org/10.64898/2026.04.25.720833; this version posted April 29, 2026. The copyright holder for this preprint (which was not certified by peer review) is the author/funder, who has granted bioRxiv a license to display the preprint in perpetuity. It is made available under aCC-BY 4.0 International license.

input in place of a file path.[1] Because SpikeInterface provides recording extractors for a wide range of electrophysiology file formats, including Open Ephys, Intan, Neuralynx, Plexon, Blackrock, and others, this indirectly extends the module's format support to the full SpikeInterface ecosystem.

Two entry points handle different recording scenarios. `sort_recording()` accepts a list of recording file paths and sorts each independently. When a directory is passed instead of a file, all recordings in the directory are concatenated into a single continuous trace (using alphabetic ordering of file names), sorted together, and split back into per-file SpikeData objects with epoch-specific waveform templates. `sort_multistream()` handles multi-well Maxwell recordings by iterating over user-specified well identifiers (stream IDs) and sorting each well independently. Similarly, passing a directory of multi-well recordings will concatenate the different recordings before sorting, at a well by well level.

Both entry points support time-windowed sorting: a single contiguous window (`start_time_s`, `end_time_s`), the first N minutes of a recording (`first_n_mins`), or multiple disjoint windows (`rec_chunks_s`) that are concatenated before sorting and split afterward.

Curation

Curation is optional and can be disabled entirely, applied during sorting with configurable thresholds, or performed post-hoc on existing SpikeData objects. The pipeline supports five curation criteria: minimum firing rate (default: 0.05 Hz), maximum ISI violation ratio (default: 1.0%, computed at a 1.5 ms threshold), minimum signal-to-noise ratio (default: 5.0), minimum spike count (default: 50), and maximum normalized waveform standard deviation (default: 1.0), which removes units with spatially inconsistent waveform shapes. All thresholds are configurable through a `CurationConfig` dataclass. For concatenated recordings, curation can be restricted to a single epoch, allowing quality assessment on the most representative segment while retaining spikes across the full recording.

When curation is applied, the pipeline produces a serializable curation history that records: the initial unit set, each criterion with its threshold and per-unit pass/fail outcome, per-unit quality metrics, and the final curated unit set. This history is saved as JSON alongside the results. Post-hoc curation methods on SpikeData objects allow researchers to apply additional or alternative filters after sorting without rerunning the pipeline. Each method returns a new SpikeData object and per-unit metric arrays, preserving the original data.

SpikeData construction and provenance

The pipeline converts sorted waveform data into SpikeData objects with full provenance metadata. Each SpikeData object stores spike trains in milliseconds (consistent with the library convention) along with per-unit neuron attributes and recording-level metadata. Recording-level metadata includes the source file path, sorting backend identifier, sampling frequency, electrode positions, and in case of concatenated recordings, epoch boundaries in both samples and milliseconds with the original file names. This metadata is sufficient to trace any SpikeData object back to its source recording and sorting parameters.

bioRxiv preprint doi: https://doi.org/10.64898/2026.04.25.720833; this version posted April 29, 2026. The copyright holder for this preprint (which was not certified by peer review) is the author/funder, who has granted bioRxiv a license to display the preprint in perpetuity. It is made available under aCC-BY 4.0 International license.

Per-unit neuron attributes include: the original cluster ID from the sorter, the maximum-amplitude channel and its electrode coordinates, waveform templates (1D on the max channel, 2D across all channels, and a windowed version), peak amplitude and per-channel amplitudes, signal-to-noise ratio, normalized waveform standard deviation, waveform polarity (positive or negative peak), and the raw spike train in samples for lossless round-tripping. For concatenated recordings, per-epoch average templates are stored separately, enabling drift assessment across recording segments.

The resulting SpikeData objects are directly compatible with all downstream analysis methods described in the main text: firing rate computation, event-aligned slicing, pairwise comparison matrices, population coupling, and latent variable models. This closes the loop between raw recordings and the analysis pipeline.

Quality control figures
The pipeline optionally generates diagnostic figures at two stages. Before curation, per-unit figures are generated for every detected unit containing an ISI histogram (0–100 ms), a spatial waveform footprint across electrode positions, and a max-channel overlay showing individual spike traces with the mean waveform. After curation, these figures are sorted into `curated/` and `failed/` subdirectories, providing a visual audit trail for every curation decision.

Post-curation figures include: a bar plot comparing total versus curated unit counts, a scatter plot of normalized waveform STD versus spike count with curation threshold lines, stacked waveform templates grouped by polarity, four-panel quality metric histograms (SNR, firing rate, spike count, ISI violations. All distributions are computed on the full pre-curation population with threshold lines overlaid), and a raster plot with population rate for the first 30 seconds of the recording.

Stimulation-aware sorting
For experiments involving electrical stimulation of neural tissue, the module provides a two-step workflow: propagation sequences are first detected in an intrinsic activity recording using RT-Sort[4] and then applied to sort spikes in the artifact-contaminated stimulation recording.

In Step 1, a baseline recording of intrinsic (unstimulated) activity is sorted using RT-Sort (or a period of the stimulation recording with no stimulations). The resulting RT-Sort object, containing detected propagation sequence templates, is serialized to disk. In Step 2, `sort_stim_recording()` processes the stimulation recording using the pre-trained sequences. This function first recenters logged stimulation times to actual artifact peaks in the voltage traces (correcting for timing jitter between the stimulation controller and the recording system), then removes stimulation artifacts using per-event polynomial detrending adapted from SALPA [Wagenaar & Potter, J Neurosci Methods 2002]. The polynomial fit captures the slow artifact waveform while preserving fast spike waveforms that co-occur with the artifact. The cleaned traces are then sorted using the pre-detected RT-Sort sequences, and spikes are aligned to the corrected stimulation times.

bioRxiv preprint doi: https://doi.org/10.64898/2026.04.25.720833; this version posted April 29, 2026. The copyright holder for this preprint (which was not certified by peer review) is the author/funder, who has granted bioRxiv a license to display the preprint in perpetuity. It is made available under aCC-BY 4.0 International license.

The output is a SpikeSliceStack aligned to stimulation events, enabling direct analysis of stimulus-evoked responses with the same event-aligned methods used throughout the library. Sequential stimulation protocols (e.g., burst stimulation, paired-pulse) are handled by dynamically extending the blanking region around each stimulation event. Individual pipeline components (artifact time recentering and artifact removal) are also available as standalone functions for custom workflows.

Spike-sorter skill

The spike-sorter skill wraps the sorting module for agentic access, extending the text-to-analysis framework from the analysis stage (covered by the analysis-implementer skill) back to the data preprocessing stage. The top-level SpikeLab skill automatically routes spike sorting requests to the spike-sorter skill based on the user's natural language prompt so that users do not need to select the correct skill manually. The skill operates under the same bounded autonomy constraints as the analysis-implementer.

File boundaries: Raw recording files are read-only. The skill never modifies, moves, or deletes source data. Sorting scripts and results are written to a user-confirmed results directory, never to the library source tree.

Analysis boundaries: The skill is restricted to sorting and output quality assessment (unit counts, SNR and firing rate distributions, waveform template inspection, and curation outcomes). For any downstream analysis, the skill directs the user to the analysis-implementer skill with the curated SpikeData pickle as the handoff point.

Clarification-seeking: The skill does not assume recording formats, electrode configurations, sorter choices, or curation thresholds. It asks the user to confirm: the recording format, whether the experiment involves electrical stimulation, single versus multi-well layout, the sorting backend, and any non-default curation parameters.

Mandatory reporting: After every sorting run, the skill generates a markdown sorting report with the curation outcome (raw versus curated unit counts, total spikes, mean firing rate, mean SNR) at the top, followed by pipeline settings, stage-by-stage timing, and unit quality distributions. This report, combined with the curation history JSON and quality control figures, provides a complete audit trail from raw recording to curated SpikeData.

Downstream handoff: The curated SpikeData pickle (`sorted_spikedata_curated.pkl`) serves as the bridge between the spike-sorter and analysis-implementer skills. A user can sort recordings in one session and load the results for analysis in a subsequent session, with full provenance metadata preserved. This two-skill design separates spike sorting (where parameter choices depend on the recording hardware and tissue type) from analysis (where method choices depend on the scientific question), while maintaining a single data structure, SpikeData, as the interface between them.

bioRxiv preprint doi: https://doi.org/10.64898/2026.04.25.720833; this version posted April 29, 2026. The copyright holder for this preprint (which was not certified by peer review) is the author/funder, who has granted bioRxiv a license to display the preprint in perpetuity. It is made available under aCC-BY 4.0 International license.

Batch-jobs module

SpikeLab includes a batch jobs module (`spikelab.batch_jobs`) that submits analysis and spike sorting workloads to remote Kubernetes clusters. The module is an optional dependency (`pip install spikelab[batch-jobs]`) and is intended for compute-intensive workflows, like long-running spike sorting, large parameter sweeps or fitting latent-variable models, that exceed the resources of a typical analysis workstation. A natural-language instruction file (`INSTRUCTIONS.md`) ships with the module, allowing the analysis-implementer and spike-sorter skills to deploy jobs on the user's behalf without leaving the text-to-analysis workflow.

Submission modes

Three submission entry points cover the common deployment patterns:

`submit_workspace_job()` saves an `AnalysisWorkspace` to disk, bundles it with a user-supplied analysis script, uploads the bundle to S3-compatible storage, and submits a Job that re-loads the workspace, runs the script, and writes the updated workspace back to S3.

`submit_sorting_job()` bundles a list of recording paths together with a `SortingPipelineConfig` (or a preset name) and runs the spike sorting pipeline (Supplementary materials: Spikesorting module) on the cluster.

`submit_prepared_job()` submits a Job without bundling artifacts, for users who manage their own input/output paths. All three modes return a `SubmitResult` containing the job name, rendered manifest, run identifier, and S3 prefixes for outputs and logs.

The complementary `retrieve_result()` method downloads the outputs after completion and reconstructs an `AnalysisWorkspace`. For sorting jobs, each recording's curated SpikeData is loaded into a separate workspace namespace, preserving the per-recording structure of the input.

Cluster profiles

Cluster-specific configuration is decoupled from job specifications through the `ClusterProfile` model. A profile bundles namespace, default CPU/GPU images, S3 prefix, namespace hooks (credential mounts and environment variables), affinity/tolerations, storage path templates, and policy thresholds. A generic `defaults` profile ships with the package, and users can supply custom profiles as YAML files via `--profile-file`, allowing the same job spec to be deployed to different clusters by switching profiles only. Namespace hooks automatically mount Kubernetes secrets and inject credential environment variables (e.g., `AWS_SHARED_CREDENTIALS_FILE`, `KUBECONFIG`), so containers do not need to be root or carry baked-in credentials.

Policy preflight

bioRxiv preprint doi: https://doi.org/10.64898/2026.04.25.720833; this version posted April 29, 2026. The copyright holder for this preprint (which was not certified by peer review) is the author/funder, who has granted bioRxiv a license to display the preprint in perpetuity. It is made available under aCC-BY 4.0 International license.

Every submission runs through a configurable policy engine (`evaluate_policy`) before the manifest is rendered. The default rules cap interactive GPU count, cap maximum runtime (default: 14 days), warn when CPU/memory requests and limits diverge, and block batch commands that resemble idle placeholders (`sleep infinity`, bare `sleep`, `sleep <large-number>`). Findings are tagged `PASS`, `WARN`, or `BLOCK`; a `BLOCK` raises `RuntimeError` unless the caller passes `allow_policy_risk=True`. Thresholds are read from the active profile, so different clusters can enforce different rules without code changes.

Artifact packaging and storage

Bundles are deterministic ZIP archives produced by `package_analysis_bundle()`, which records SHA-256 hashes for every input file in a manifest alongside the bundle. Bundles, outputs, and logs are addressed through the profile's `StoragePathTemplates` (`{prefix}/inputs/{run_id}/...`, `{prefix}/outputs/{run_id}/...`, `{prefix}/logs/{run_id}/...`), giving each run a self-contained S3 namespace that can be re-downloaded, archived, or shared. The `S3StorageClient` works with any S3-compatible endpoint via the profile's `endpoint_url` and `region_name`, including non-AWS object stores.

CLI and Python API

The same operations are exposed through both a CLI (`spikelab-batch-jobs`) and a Python API. The CLI provides `render-job` (dry-render a manifest for inspection), `deploy-job` (submit, optionally with `--wait` and log streaming), `job-status`, `job-logs`, and `job-delete`. The Python API exposes the underlying classes (`RunSession`, `ClusterProfile`, `JobSpec`, `ContainerSpec`, `ResourceSpec`, `VolumeMountSpec`) for programmatic submission from analysis scripts. CLI and API share the same profile loader, policy engine, and manifest renderer, so behaviour is identical regardless of entry point.

Agentic integration

The module ships an `INSTRUCTIONS.md` file that describes the fixed deployment workflow (preflight checks, dry render, submission, observation, failure triage, teardown) together with credential handling rules and a first-time setup walkthrough. The analysis-implementer and spike-sorter skills load this INSTRUCTIONS.md file when the user's prompt mentions remote/cluster execution (e.g., "deploy to cluster", "submit a batch job") and follow the workflow under the same bounded autonomy constraints as their local-execution behaviour: raw recording files remain read-only, secrets are never echoed into chat or written to bundle metadata, image tags and namespaces are confirmed with the user before submission, and `--allow-policy-risk` is never used unless the user explicitly requests it. This extends the text-to-analysis framework from the local workstation to remote clusters without requiring researchers to learn `kubectl`, manage manifests by hand, or context-switch between tools.

Benchmarking examples

This supplementary section provides code examples and detailed descriptions for each issue in the benchmark scorecard (Fig. 2D). All code is extracted verbatim from the generated scripts.

bioRxiv preprint doi: https://doi.org/10.64898/2026.04.25.720833; this version posted April 29, 2026. The copyright holder for this preprint (which was not certified by peer review) is the author/funder, who has granted bioRxiv a license to display the preprint in perpetuity. It is made available under aCC-BY 4.0 International license.

See Supplementary Data (https://doi.org/10.5281/zenodo.19776254) for all generated scripts. File paths below are relative to the `benchmark/` directory inside Supplementary Data, with scripts under `raw_outputs/scripts/{condition}/{run}/`, figures under `raw_outputs/figures/{condition}/{run}/`, and anonymized transcripts under `raw_outputs/transcripts/` (renamed as `{condition}_{run}.txt`, where condition ∈ {opus, sonnet, spikelab}).

Task 1: Data Discovery

Session selection: API endpoint divergence

The IBL ONE API offers two search endpoints indexed from different sources: `one.search()` queries session-level metadata tags, while `one.search_insertions()` queries histologically verified probe trajectory data. The 208-unit session's auditory cortex coverage was documented only in trajectory metadata, not in session-level tags. Sonnet queried all four auditory subregions and pooled the results but used `one.search()`; Opus discovered `one.search_insertions()` after its initial approach failed.

Sonnet, session-level search across 4 auditory subregions, pooled and deduplicated:

```
AUD_ACRONYMS = ['AUDp', 'AUDv', 'AUDd', 'AUDpo']
all_eids = []
for acr in AUD_ACRONYMS:
    eids = one.search(atlas_acronym=acr)
    all_eids.extend(eids)
all_eids = list(dict.fromkeys(all_eids))  # deduplicate
```

Source: `raw_outputs/scripts/sonnet_plain/260324/ibl_auditory_raster.py:23–38`

Opus, trajectory-level search, which returns the correct session:

```
insertions = one.search_insertions(atlas_acronym='AUD')
```

Source: `raw_outputs/scripts/opus_plain/250324/find_session4.py`

SpikeLab, downloads the complete Brain-Wide Map unit table, sidestepping endpoint gaps:

```
probes, stats = query_ibl_probes(
    target_regions=AUDITORY_REGIONS, min_units=1)
stats_sorted = stats.sort_values("n_in_target", ascending=False)
best = stats_sorted.iloc[0]
```

Source: `raw_outputs/scripts/sonnet_spikelab/260324/plot_auditory_raster.py:57–61`

Data caching

bioRxiv preprint doi: https://doi.org/10.64898/2026.04.25.720833; this version posted April 29, 2026. The copyright holder for this preprint (which was not certified by peer review) is the author/funder, who has granted bioRxiv a license to display the preprint in perpetuity. It is made available under aCC-BY 4.0 International license.

SpikeLab, cached data to pickle on first load and stored intermediate results in HDF5 workspace files:

```
ws = AnalysisWorkspace.load(WS_PATH)
sd = ws.get("recording", "spikedata")          # raw data
z_sttc = ws.get("sttc", "z_sttc_cue")          # intermediate result
coupling = ws.get("stpr", "coupling_zero_lag")   # intermediate result
```

Source: `raw_outputs/scripts/sonnet_spikelab/260326/compute_sttc.py:171` (save), `raw_outputs/scripts/sonnet_spikelab/260326/plot_stpr_raster.py:35–37` (load)

Opus, (run 260325 only) saved raw arrays but no intermediate results:

```
spike_times = np.load('spike_times.npy')
spike_clusters = np.load('spike_clusters.npy')
```

Source: `raw_outputs/scripts/opus_plain/260325/pca_hmm_analysis.py:19–20`

Sonnet, never cached data or intermediate results in any run.

Project organization

SpikeLab, created structured directories:

```
os.makedirs(FIGURES_DIR, exist_ok=True)
os.makedirs(os.path.join(ANALYSIS_DIR, "results"), exist_ok=True)
```

Source: `raw_outputs/scripts/sonnet_spikelab/260326/compute_sttc.py:31–32`

Sonnet and Opus, produced flat directories with throwaway scripts (e.g., Opus `find_session.py` through `find_session5.py`, `check_trials.py`, `check_trials2.py` in run 250324).

Task 2: STTC with Shuffle Controls

Clarification-seeking

SpikeLab asked for clarification in all three runs:

What do you mean by "cue" vs "no-cue" parts of the recording?

A few possible interpretations:
1. Cue period = stimulus on window

bioRxiv preprint doi: https://doi.org/10.64898/2026.04.25.720833; this version posted April 29, 2026. The copyright holder for this preprint (which was not certified by peer review) is the author/funder, who has granted bioRxiv a license to display the preprint in perpetuity. It is made available under aCC-BY 4.0 International license.

2. Cue period = go-cue aligned window
3. Cue = stimulus-present trials vs catch trials
4. Cue = all stimulus-present intervals concatenated vs complement
Which of these did you have in mind?

Source: `raw_outputs/transcripts/spikelab_260326.txt:428–443`

Neither Opus nor Sonnet asked for clarification in any of their six runs. Both proceeded directly to implementation, silently choosing an interpretation. Opus consistently defined cue as stimulus onset to feedback, and no-cue as inter-trial intervals, across all three runs:

```
cue_intervals = np.column_stack([trials['stimOn_times'], trials['feedback_times']])
nocue_intervals = np.column_stack([trial_ends[:-1], trial_starts[1:]])
```

Source: `raw_outputs/scripts/opus_plain/260326/sttc_analysis.py:174–183`

Sonnet used a different operationalization in each of its three runs, producing three fundamentally different experimental designs without flagging any of them as a choice.

260324: Trial-type split. Cue = stimOn→stimOff for non-zero contrast trials. No-cue = stimOn→stimOff for zero-contrast (catch) trials.

```
cue_ivs = np.column_stack([stim_on[valid &  has_stim], stim_off[valid &  has_stim]])
nocue_ivs = np.column_stack([stim_on[valid & ~has_stim], stim_off[valid & ~has_stim]])
```

Source: `raw_outputs/scripts/sonnet_plain/260324/ibl_sttc_analysis.py:79–82`

260325: Matched-duration peri-event windows. Cue = [goCue, goCue + 1 s]. No-cue = [goCue − 2 s, goCue − 1 s].

```
cue_ws = cue_t
cue_we = cue_t + 1.0
nocue_ws = cue_t - 2.0
nocue_we = cue_t - 1.0
```

Source: `raw_outputs/scripts/sonnet_plain/260325/ibl_sttc.py:62–68`

260326: Temporal split (same as Opus). Cue = stimOn→stimOff for all trials. No-cue = Inter-trial intervals.

```
cue_ivs = np.column_stack([stim_on[valid], stim_off[valid]])
iti_starts = ivs[:-1, 1]
iti_ends   = ivs[1:,  0]
nocue_ivs  = np.column_stack([iti_starts[nocue_valid], iti_ends[nocue_valid]])
```

bioRxiv preprint doi: https://doi.org/10.64898/2026.04.25.720833; this version posted April 29, 2026. The copyright holder for this preprint (which was not certified by peer review) is the author/funder, who has granted bioRxiv a license to display the preprint in perpetuity. It is made available under aCC-BY 4.0 International license.

Source: `raw_outputs/scripts/sonnet_plain/260326/ibl_sttc.py:65–73`

Each of these is a valid interpretation of the prompt, but they answer different scientific questions. A researcher receiving these results across runs would not be able to compare them without realizing they measure different things.

Shuffle issues

Opus remapped spike times to a continuous timeline containing only cue intervals. However, when applying circular shuffling after collapsing all trials into a single stream, trial-to-trial firing rate variability got destroyed:

```
def flatten_trains(trains):
    offsets = np.zeros(len(trains) + 1, dtype=np.int64)
    for i, t in enumerate(trains):
        offsets[i + 1] = offsets[i] + len(t)
    flat = np.concatenate(trains)
    return flat, offsets

def shuffle_trains(trains, total_time, rng):
    """Circular shuffle each spike train independently."""
    shuffled = []
    for t in trains:
        if len(t) == 0:
            shuffled.append(t.copy())
            continue
        shift = rng.uniform(0, total_time)
        shifted = np.mod(t + shift, total_time)
        shifted.sort()
        shuffled.append(shifted)
    return shuffled
```

Source: `raw_outputs/scripts/opus_plain/260325/sttc_analysis.py:181–201`

Sonnet also remapped spike times and introduced a 40ms window between consecutive cue intervals. Subsequent circular shuffling did not just destroy trial-to-trial firing rate variability but also caused shuffled spikes to leak into the empty gap windows between trials:

```
def compress_spikes(spike_times, intervals, gap=2 * DELTA):
    ivs = intervals[np.argsort(intervals[:, 0])]
    parts  = []
    offset = 0.0
    for start, end in ivs:
```

bioRxiv preprint doi: https://doi.org/10.64898/2026.04.25.720833; this version posted April 29, 2026. The copyright holder for this preprint (which was not certified by peer review) is the author/funder, who has granted bioRxiv a license to display the preprint in perpetuity. It is made available under aCC-BY 4.0 International license.

```
        dur  = end - start
        mask = (spike_times >= start) & (spike_times < end)
        parts.append(spike_times[mask] - start + offset)
        offset += dur + gap
    total = max(offset - gap, 1e-6)
    arr   = np.concatenate(parts) if parts else np.array([])
    return np.sort(arr), total

def circular_shift(a, total):
    """Circular shift spike train by a random amount >= MIN_SHIFT."""
    shift = RNG.uniform(MIN_SHIFT, total - MIN_SHIFT)
    return np.sort((a + shift) % total)
```

Source: `raw_outputs/scripts/sonnet_plain/260324/ibl_sttc_analysis.py:90–107, 155–158`

SpikeLab applied a degree-preserving shuffle method from Okun et al.[6] which ensured consistent spikes counts for each time frame before and after shuffling.

```
shuf_stack = sd_cond.spike_shuffle_stack(n_shuffles=10, seed=42)
shuf_mat = shuf_stack.apply(lambda sd: sd.spike_time_tilings(delt=20).matrix)
```

Source: `raw_outputs/scripts/sonnet_spikelab/260326/compute_sttc.py:155–158`

Near-zero variance clamping

Sonnet hit a numerical edge case for sparse pairs. The fix applied arbitrary clamping:

```
zval = np.clip((r - mu) / max(sd, MIN_SD), -Z_CLIP, Z_CLIP)
```

Source: `raw_outputs/scripts/sonnet_plain/260324/ibl_sttc_analysis.py:195–196`

The clamping strategy was not reported and the clamping threshold varied across runs:

260324 & 260326: Arbitrary floor of `MIN_SD = 5e-3`, `Z_CLIP = 10.0`
260325: Principled floor of theoretical Poisson sigma = `sqrt(2*delta/T_tot)`

Task 3: Population Coupling

Opus method drift across runs

Opus invented a different coupling formula in each run:

260324: Dimensionless z-score

bioRxiv preprint doi: https://doi.org/10.64898/2026.04.25.720833; this version posted April 29, 2026. The copyright holder for this preprint (which was not certified by peer review) is the author/funder, who has granted bioRxiv a license to display the preprint in perpetuity. It is made available under aCC-BY 4.0 International license.

```
coupling = (mean_pop_at_spikes - mean_pop_overall) / std_pop_overall
```

Source: `opus_plain/250324/pop_coupling.py`

260325: Unitless ratio

```
coupling = stpr_zero_lag / mean_pop_rate
```

Source: `opus_plain/260325/population_coupling.py`

260326: Raw firing rate (spk/s)

```
coupling = stpr_zero_lag - baseline_rate
```

Source: `opus_plain/260326/population_coupling.py`

Sonnet consistently applied the population coupling computations from Okun et al.[7]:

```
for k in range(N):
    loo_rate = pop_rate - unit_smooth[k]    # leave-one-out

    spike_bins = np.where(unit_counts[k] > 0)[0]
    if unit_counts[k].max() > 1:
        spike_bins = np.repeat(spike_bins,
                               unit_counts[k][spike_bins].astype(int))

    spike_bins = spike_bins[(spike_bins >= WIN_BINS) &
                            (spike_bins < n_bins - WIN_BINS)]
    if len(spike_bins) == 0:
        continue

    idx_mat = spike_bins[:, None] + np.arange(-WIN_BINS, WIN_BINS + 1)
    windows = loo_rate[idx_mat]
    stpr_all[k] = windows.mean(axis=0) / (BIN * (N - 1))

    zero_lag_coupling[k] = stpr_all[k, WIN_BINS]
```

Source: `raw_outputs/scripts/sonnet_plain/260324/ibl_stpr_psth.py:105–127`

SpikeLab consistently applied the population coupling computations with normalization from Bimbard et al.[8] via a library call:

```
stpr, coupling_zero_lag, coupling_max, delays, lags = \
    sd_aud.compute_spike_trig_pop_rate()
```

Source: `raw_outputs/scripts/sonnet_spikelab/260324/plot_stpr_raster_psth.py:63–64`

bioRxiv preprint doi: https://doi.org/10.64898/2026.04.25.720833; this version posted April 29, 2026. The copyright holder for this preprint (which was not certified by peer review) is the author/funder, who has granted bioRxiv a license to display the preprint in perpetuity. It is made available under aCC-BY 4.0 International license.

This yielded identical results across all three runs: coupling range -0.14 to +0.43, median 0.066, selected unit index 35 (AUDd, 1.85 Hz).

Task 4: Latent State Manifold

Silent scope reduction

Sonnet applied downsampling with no stated reasoning:

```
HMM_STEP = 2    # run 260324
DS_FACTOR = 4   # run 260326
```

Source: `raw_outputs/scripts/sonnet_plain/260324/ibl_pca_hmm.py:28`, `raw_outputs/scripts/sonnet_plain/260326/ibl_pca_hmm.py:33`

Opus (run 260325) killed a stuck HMM process and rewrote with coarser parameters:

"The HMM fitting with 93k timepoints and multiple restarts is slow. Let me check if the process is still alive, and if so, kill it and rewrite with a faster approach."
Source: `raw_outputs/transcripts/opus_260325.txt:428–438`

SpikeLab consistently used the full recording:

```
gplvm_result = sd_aud.fit_gplvm(bin_size_ms=50)
```

Source: `raw_outputs/scripts/sonnet_spikelab/260324/compute_pca_gplvm.py:114`

BIC boundary problem

Both Sonnet and Opus tried a range of hidden state counts for their HMM model fitting and used BIC to select the optimal number of hidden states. However, both selected the maximum number of hidden states in the range in 2 of 3 runs, without extending the search range:

```
bic_scores = {}
models = {}
for K in range(2, 8):
    m = GaussianHMM(n_components=K, covariance_type='diag',
                    n_iter=200, tol=1e-4, random_state=42)
    m.fit(X_fit)
    ll = m.score(X_fit) * len(X_fit)
    n_par = K*(K-1) + (K-1) + 2*K*D       # free params (diag cov)
    bic = -2*ll + n_par * np.log(len(X_fit))
```

bioRxiv preprint doi: https://doi.org/10.64898/2026.04.25.720833; this version posted April 29, 2026. The copyright holder for this preprint (which was not certified by peer review) is the author/funder, who has granted bioRxiv a license to display the preprint in perpetuity. It is made available under aCC-BY 4.0 International license.

```
    bic_scores[K] = bic
    models[K]     = m

best_K = min(bic_scores, key=bic_scores.get)  # selected 7 (max tested)
```

Source: `raw_outputs/scripts/sonnet_plain/260325/ibl_pca_hmm.py:94–110`

```
n_states_range = range(2, 9)
best_bic = np.inf
best_n = 2

for n_states in n_states_range:
    model = GaussianHMM(n_components=n_states, covariance_type='full',
                        n_iter=200, random_state=42)
    model.fit(hmm_input)
    log_likelihood = model.score(hmm_input) * len(hmm_input)
    n_params = (n_states * n_pcs_for_hmm +
               n_states * n_pcs_for_hmm * (n_pcs_for_hmm + 1) // 2 +
               n_states * (n_states - 1) + n_states - 1)
    bic = -2 * log_likelihood + n_params * np.log(len(hmm_input))
    if bic < best_bic:
        best_bic = bic
        best_n = n_states
```

Source: `raw_outputs/scripts/opus_plain/250324/pca_ssm.py:117–144`

In both cases, when the selected K equals the upper bound of the search range, the standard practice is to extend the range to verify the BIC minimum is genuine rather than an artifact of the truncated search. Neither model applied this check. SpikeLab sidesteps this problem entirely by using a GPLVM instead of an HMM.[9] The GPLVM determines effective dimensionality from the data via the GP prior rather than requiring discrete state count selection so there is no K parameter to search over and therefore no boundary to hit.

Global: Workflow Behaviors

Analysis logging

SpikeLab created and maintained `ANALYSIS_LOG.md` after every task:

```
# Analysis Log — IBL Auditory Cortex

## Experiment Context
Data source: IBL Brain-Wide Map (public ONE API).
```

bioRxiv preprint doi: https://doi.org/10.64898/2026.04.25.720833; this version posted April 29, 2026. The copyright holder for this preprint (which was not certified by peer review) is the author/funder, who has granted bioRxiv a license to display the preprint in perpetuity. It is made available under aCC-BY 4.0 International license.

Goal: Identify the IBL probe with the most good units in
auditory cortex, download it, and visualise activity.

Source: `raw_outputs/scripts/sonnet_spikelab/260324/ANALYSIS_LOG.md:1–6`

Neither Opus nor Sonnet kept a log of the analyses.

Caveat flagging

SpikeLab proactively flagged limitations:

Caveats:
- ITI total duration (282 s) is ~4x shorter than cue (1,239 s),
  making z-score estimates noisier for the no-cue condition.
- The spike_shuffle operates on the full remapped train, not
  per-trial; this mixes within- and across-trial shuffling.

Source: `raw_outputs/scripts/sonnet_spikelab/260324/ANALYSIS_LOG.md:62–64`

Neither Opus nor Sonnet reported caveats back to the user.

Follow-up suggestions

SpikeLab proactively suggested follow up analyses:

Open Questions
- The two auditory subregions (AUDd, AUDv) could be compared
  separately.
- Should the 409 outlier pairs be characterised?
- Follow-up: PCA/UMAP on the lower-triangle z-STTC vectors to
  see if cue vs. no-cue separate in low-dimensional space.

Source: `raw_outputs/scripts/sonnet_spikelab/260324/ANALYSIS_LOG.md:116–122`

Neither Opus nor Sonnet suggested follow-ups to the user.

Supplementary references

1. Buccino, A. P., Hurwitz, C. L., Garcia, S., Magland, J., Siegle, J. H., Hurwitz, R., & Hennig, M. H. (2020). SpikeInterface, a unified framework for spike sorting. *Elife*, 9, e61834. https://doi.org/10.7554/eLife.61834

bioRxiv preprint doi: https://doi.org/10.64898/2026.04.25.720833; this version posted April 29, 2026. The copyright holder for this preprint (which was not certified by peer review) is the author/funder, who has granted bioRxiv a license to display the preprint in perpetuity. It is made available under aCC-BY 4.0 International license.

2. Pachitariu, M., Steinmetz, N., Kadir, S., Carandini, M. & Harris K. D. Kilosort: realtime spike-sorting for extracellular electrophysiology with hundreds of channels. bioRxiv https://doi.org/10.1101/061481 (2016).

3. Pachitariu, M., Sridhar, S., Pennington, J., & Stringer, C. (2024). Spike sorting with Kilosort4. *Nature methods*, 21(5), 914-921. https://doi.org/10.1038/s41592-024-02232-7

4. Van der Molen, T., Lim, M., Bartram, J., Cheng, Z., Robbins, A., Parks, D. F., ... & Kosik, K. S. (2024). RT-Sort: An action potential propagation-based algorithm for real time spike detection and sorting with millisecond latencies. PloS one, 19(12), e0312438.

5. Wagenaar, D. A., & Potter, S. M. (2002). Real-time multi-channel stimulus artifact suppression by local curve fitting. Journal of neuroscience methods, 120(2), 113-120.

6. Okun, M., Yger, P., Marguet, S. L., Gerard-Mercier, F., Benucci, A., Katzner, S., ... & Harris, K. D. (2012). Population rate dynamics and multineuron firing patterns in sensory cortex. *Journal of Neuroscience,* 32(48), 17108-17119. https://doi.org/10.1523/JNEUROSCI.1831-12.2012

7. Okun, M., Steinmetz, N. A., Cossell, L., Iacaruso, M. F., Ko, H., Barthó, P., ... & Harris, K. D. (2015). Diverse coupling of neurons to populations in sensory cortex. *Nature*, 521(7553), 511-515. https://doi.org/10.1038/nature14273

8. Bimbard, C., Harris, K. D., & Carandini, M. (2025). Invariant activity sequences across the mouse brain. *bioRxiv*, 2025-12. https://doi.org/10.64898/2025.12.20.695676

9. Zheng, Z., Zutshi, I., Huszár, R., Zhang, Y., Karadas, M., Buzsáki, G., & Williams, A. H. (2025). From labels to latents: revealing state-dependent hippocampal computations with Jump Latent Variable Model. *bioRxiv*, 2025-12. https://doi.org/10.64898/2025.12.14.694183
